# SGLT2 Inhibitors and Kidney Outcomes in Patients with Chronic Kidney Disease

**DOI:** 10.3390/jcm9092723

**Published:** 2020-08-24

**Authors:** Swetha R. Kanduri, Karthik Kovvuru, Panupong Hansrivijit, Charat Thongprayoon, Saraschandra Vallabhajosyula, Aleksandra I. Pivovarova, Api Chewcharat, Vishnu Garla, Juan Medaura, Wisit Cheungpasitporn

**Affiliations:** 1 Department of Nephrology, Ochsner Medical Center, New Orleans, LA 70121, USA; svetarani@gmail.com (S.R.K.); karthik.kovvuru@ochsner.org (K.K.); 2 Department of Internal Medicine, University of Pittsburgh Medical Center Pinnacle, Harrisburg, PA 17105, USA; hansrivijitp@upmc.edu; 3 Division of Nephrology and Hypertension, Mayo Clinic, Rochester, MN 55905, USA; charat.thongprayoon@gmail.com (C.T.); chewcharat.api@mayo.edu (A.C.); 4 Section of Interventional Cardiology, Division of Cardiovascular Medicine, Department of Medicine, Emory University School of Medicine, Atlanta, GA 30322, USA; saraschandra.vallabhajosyula@emory.edu; 5 Division of Nephrology, Department of Medicine, University of Mississippi Medical Center, Jackson, MS 39156, USA; apivovarova@umc.edu (A.I.P.); jmedaura@umc.edu (J.M.); 6 Department of Internal Medicine and Mississippi Center for Clinical and Translational Research, University of Mississippi Medical Center, Jackson, MS 39156, USA; vgarla@umc.edu

**Keywords:** Diabetes mellitus, SGLT2, SGLT2i, sodium glucose co-transporter 2 inhibitors, nephrology, endocrinology, cardiology

## Abstract

Globally, diabetes mellitus is a leading cause of kidney disease, with a critical percent of patients approaching end-stage kidney disease. In the current era, sodium-glucose co-transporter 2 inhibitors (SGLT2i) have emerged as phenomenal agents in halting the progression of kidney disease. Positive effects of SGLT2i are centered on multiple mechanisms, including glycosuric effects, tubule—glomerular feedback, antioxidant, anti-fibrotic, natriuretic, and reduction in cortical hypoxia, alteration in energy metabolism. Concurrently, multiple kidney and cardiovascular outcome studies have reported remarkable advantages of SGLT2i including mortality benefits. Additionally, the superiority of combination therapies (SGLT2I along with metformin/DDP-4 Inhibitors) in treatment-naïve diabetic patients is further looked into with potential signal towards glycemic and blood pressure control. Reported promising results initiate a gateway for future research targeting kidney outcomes with combination therapies as an initial approach. In the current paper, we summarize leading cardiovascular and kidney outcome trials in patients with type 2 diabetes, the role of SGLT2i in non-diabetic proteinuric kidney disease, and the potential mechanisms of action of SGLT2i with special focus on combination therapy as an initial therapeutic approach in treatment-naïve diabetic patients.

## 1. Introduction

Diabetes mellitus and associated conditions including hypertension, obesity, and atherosclerosis significantly contribute to progression of chronic kidney disease (CKD), cardiovascular health, and overall mortality [1,2,3,4,5,6,7,8]. Diabetes is an evolving global pandemic with diabetic kidney disease accounting for 44.5% of new end-stage kidney disease (ESKD) cases [1,2]. Two defined pathways that have been proposed to describe the evolution of diabetic kidney disease are hemodynamic and non-hemodynamic [9]. Although not fully understood, the role of hyperglycemia in pathophysiology of diabetic complications has been attributed to an increase in intra-glomerular pressure, elevation of single nephron glomerular filtration rate (GFR), and podocyte damage further perpetuating renal dysfunction [10]. Other contributory mechanisms include neurohumoral activation and cytokine release, along with proinflammatory pathway activation, potentiating tubulointerstitial inflammation and fibrosis [11,12].

Over the past 20 years, angiotensin receptor blockers (ACE) have been used in attenuating neuro-humoral activation and reducing intra-glomerular hypertension. ACE inhibitors reduce doubling of serum creatinine and progression to ESKD by about 20% [13,14]. Even though renin-angiotensin aldosterone system (RAAS) blockade helps reduce glomerular hypertension, they were unsuccessful in normalizing hyperfiltration, reduction of cardiovascular disease, and mortality [15]. With the introduction of sodium-glucose co-transporter 2 inhibitors (SGLT2i) there has been a fundamental change in treatment paradigm of patients with CKD secondary to diabetic nephropathy [16,17,18,19,20].

SGLT2i have been increasingly recognized for their remarkable renoprotective and cardioprotective benefits [21,22,23,24]. Not surprisingly, because of their well-established benefits, SGLT2i has reshaped the treatment algorithm of type 2 diabetes mellitus. After the initial discovery of phlorizin, a non-selective SGLTi, multiple other formulations have since emerged [25,26]. Approximately 80–90% of filtered glucose is actively reabsorbed via SGLT2, located at the S1 segment of the proximal tubule, at a concentration of 1:1 with sodium. Additionally, SGLT1, located at S2/S3 segment of the proximal tubule, utilizes more energy and helps to reabsorb 10–20% of glucose in association with two sodium molecules [27,28]. Because of their glycosuric properties, SGLT2i contributes to weight loss of approximately 2 to 3 kg [29,30]. Subsequently, 3 to 5 mmHg systolic and 1 to 2 mmHg diastolic blood pressure lowering effects are being encountered [29,30,31]. The above-mentioned anti-hypertensive benefits of SGLT2i are implicated across all ranges of estimated GFR (eGFR) even among patients with stage 4 CKD [31]. Multiple randomized controlled studies have reported substantial benefits of combination therapy with SGLT2i and metformin as initial approach in patients with type 2 diabetes [32,33,34]. With that being said, American Diabetes Association 2020 guidelines recommend prescribing an SGLT2i following initial trial of lifestyle modifications and metformin in patients with CKD, cardiovascular disease, and heart failure [35].

The seminal study by Milder et al. reviewed efficacy and safety of a combination approach of SGLT2i and metformin in treatment-naïve type 2 diabetic patients [36]. Four randomized controlled studies with a total of 3749 patients were included. The outcomes of the study were substantially in favor of combination therapy, showing significant reduction in hemoglobin A1c compared with monotherapy after 24–26 weeks of treatment. High dose SGLT2i/metformin combination therapy dapagliflozin 10 mg or canagliflozin 300 mg, as compared to low dose combination therapy dapagliflozin 5 mg or canagliflozin 100 mg, appears to cause modest weight reduction without glycemic benefits. Additionally, data revealed that combination therapy provided statistically significant reduction in systolic and diastolic blood pressure as compared to metformin alone. However, no difference in blood pressure was noted when combination therapy is compared to SGLT2i alone. Safety profile was in favor of combination therapy, with a mildly increased risk of diarrhea with combination therapy. Although this systematic review reported particular benefits of combination therapy as initial strategy, it did not address the role of combination therapy in lowering proteinuria or effects on rise of serum creatinine.

Significant benefits of SGLT2i in type 2 diabetic patients outweigh minor side effect profile mentioned in the literature. Furthermore, appropriate preventive steps can be undertaken to help mitigate potential adverse effects. For example, SGLT2i has been associated with increased risk of mycotic genital infections, necessitating frequent monitoring and good hygiene. It has been proposed that prophylactic antifungals could be considered in patients with high risk of fungal infections. Additionally, SGLT2i has demonstrated significant natriuretic effects, which necessitates holding the doses in patients with nausea and vomiting or other conditions that would make them more prone to dehydration. Avoiding SGLT2i with early signs of DKA is also recommended due to concerns of worsening acidosis. Careful consideration should be given to the risk of urinary tract infections associated with SGLT2i. Lastly, patients with open foot wounds or skin ulcers should also be cautious due to some existing reports of lower limb amputations linked to SGLT2i [37,38,39,40].

## 2. Clinical Trials

### 2.1. Major Cardiovascular Outcomes Trials

Based on recognized health benefits of SGLT2i, multicenter, randomized controlled studies were conducted evaluating renal and cardiovascular outcomes. As a pioneer agent, empagliflozin was compared to placebo in patients with type 2 diabetes mellitus at high risk of cardiovascular events [41]. Among 7020 diabetic patients included in the study, there were 1819 CKD patients with GFR > 30 mL/min [21]. Interestingly, a 14% reduction in risk of death, including from cardiovascular causes, non-fatal myocardial infarctions (MI), and non-fatal stroke, was reported. Wanner et al. analyzed long term renal outcomes from participants of EMPA REG OUTCOME study. The results of the study showed a 39% relative risk reduction in progression of albuminuria, a 44% relative risk reduction in serum creatinine doubling, and a 55% relative risk reduction in initiation of renal-replacement therapy (RRT) [41]. Subsequently, CANVAS trial compared efficacy and safety of canagliflozin in patients with type 2 diabetes compared to placebo [42]. This study included 10,000 participants reporting cardiovascular outcomes. Around 2039 patients had kidney disease with a mean eGFR of 76.5 mL/min. Similar results were encountered with around a 14% decrease in composite risk of death from non-fatal MI, non-fatal stroke, and cardiovascular causes. While the primary goal of the trial was to evaluate cardiovascular outcomes, significant renoprotective effect was also observed. Approximately a 40% reduction in death from kidney causes, decline in GFR, and need for RRT were reported. Another multicenter placebo-controlled trial, DECLARE—TIMI, compared dapagliflozin to placebo in patients with type 2 diabetes evaluating the cardiovascular safety, confirmed sustained benefits of SGLT2i [22]. Moreover, this trial reported remarkable kidney-specific benefits with about 40% decrease in rate of GFR decline, progression to ESKD and death from kidney causes.

### 2.2. Major Kidney-Specific Outcome Trials

Some of the renal specific outcomes were studied in multicentric, double-blind, randomized trial, CREDENCE. This study looked at the effects of canagliflozin in patients with type 2 diabetes and albuminuric CKD [43]. This has been one of the first studies which specifically included albuminuric CKD patients. Included patients had GFR of 30–90 mL per minute per 1.73 m^2^ of body-surface area and albuminuria of greater than 300 mg/g. Due to the apparent benefit of the drug observed in the study, CREDENCE trial was stopped earlier. Around 34% relative risk reduction in doubling of serum creatinine, progression to ESKD, and death from renal causes have been reported. Besides renal specific advantage, cardiovascular benefits were also observed.

Recently, another multicentric, double-blind, randomized trial, DAPA-CKD, compared dapagliflozin to placebo. This trial specifically included late-stage CKD patients with GFR above 25 and below 75 mL/min, albuminuric patients with urine albumin-to-creatinine ratio of equal or above 200 and equal or below 5000 mg/g in patients with or without diabetes. Similar to CREDENCE, this trial was also stopped early given demonstrated superior efficacy of dapagliflozin. Primary endpoint was defined as a composite of an eGFR decline of at least 50%, onset of ESKD, and death from cardiovascular or renal cause in patients with CKD regardless of presence of diabetes.

Lastly, there is currently an ongoing randomized double-blind placebo-controlled EMPA-KIDNEY trial, which was designed to evaluate safety and efficacy of empagliflozin in 5000 CKD patients with albuminuria of above 200 mg/g and eGFR 20–45 mL/min or 45–90 mL/min/1.73 m^2^ (Table 1).

### 2.3. Role of SGLT2 in Non-Diabetic CKD Patients

Several pre-clinical studies/animal models have been published evaluating the role of SGLT2i in non-diabetic CKD. However, well-defined conclusion could not be established due to conflicting results [55,56,57]. As far as clinical studies, at this point it is unclear if there is a sustained clinical benefit in cardiovascular or renal outcomes in non-diabetic patients. A pilot study by Rajasekeran et al., which included ten patients with FSGS, evaluated the effects of 8 weeks of dapagliflozin on GFR and proteinuria [58]. Dapagliflozin failed to demonstrate additional effects on body weight, proteinuria or measured GFR. A Phase 2 randomized, double-blind study by Bays et al. enrolled 376 overweight and obese non-diabetic patients to evaluate the effects of canagliflozin on body weight [59]. Even though there was significant reduction in body weight, no effect on proteinuria was observed.

Dapagliflozin was studied in a randomized, double-blind, placebo-controlled study, DIAMOND, which included proteinuric, non-diabetic CKD patients with eGFR of at least 25 mL/min/1.73 m2 [50]. A total of 53 patients included in the study had proteinuria ranging from 500 to 3500 mg per 24 h. A reduction in body weight by 1.5 kilos was observed in dapagliflozin group compared to placebo during a 6-week period, yet neither significant reduction in systolic or diastolic blood pressure nor reduction in proteinuria were detected. In addition, an acute and reversible decline in measured GFR was noted in dapagliflozin group. Long-term clinical studies in evaluating the potential benefit of SGLT2i in non-diabetic CKD patients are required before reaching a meaningful conclusion (Table 2).

## 3. Proposed Renoprotective Mechanisms of SGLT2i

### 3.1. Tubulo-Glomerular Feedback Mechanism (TGF)

It has been well recognized that SGLT2i decrease serum glucose level by increasing urinary glucose excretion [61,62,63]. Renoprotective benefits of SGLT2i, apart from glucose-lowering mechanisms, are also well established. Tubulo-glomerular feedback is, thus far, the most well-explained mechanism. Sodium hydrogen exchanger (NHE3) activity is downregulated in proximal tubule by SGLT2i, reducing sodium reabsorption. This leads to a considerable amount of sodium being delivered to macula-densa, causing afferent arteriolar vasoconstriction and further reducing renal blood flow [47,64]. SGLT2i in synergy with ACE inhibitors contribute to afferent arteriole vasoconstriction and efferent vasodilatation, reducing intra-glomerular pressure [65,66,67,68].

### 3.2. Non-TGF Mediated Mechanisms

SGLT2i potentiate natriuresis, reduce total body sodium content, and lower blood pressure (EMPA REG Outcomes Trial) [21,69]. They further potentiate reduction of interstitial volume, improve endothelial function and vascular tone [70,71,72]. SGLT2i also contributes to caloric loss with substantial improvement in insulin resistance and hemoglobin A_1C_. This effect becomes evident within the first few weeks of treatment initiation and is maintained long-term [73,74]. The role of SGLT2i on modulating RAAS is controversial; studies by Yoshimoto et al. reported no significant RAAS activation [75]. Furthermore, SGLT2i also potentiates reduction in albuminuria in diabetic kidney disease by reducing intra-glomerular pressure and podocyte stabilization [20,43,76,77].

### 3.3. Antioxidant Properties

SGLT2i exhibits antioxidant properties by reducing free radical generation. Phlorizin was initially studied by Osorio et al. and was found to reduce oxidative stress in diabetic rats by its effect on catalase and glutathione peroxidase and decrease in nitrogen-free radicles [78]. Tang et al. further reported that dapagliflozin slowed the progression of diabetic nephropathy by decreasing the production of free radical progenitors, including nicotinamide adenine dinucleotide phosphate oxidase (NOX) 4, and NOX 2 [79]. In studies of murine diabetic heart by Xue et al., empagliflozin reduced oxidative stress through activating NRF2/ARE signaling pathway in type 2 diabetic models [80].

### 3.4. Anti-Inflammatory Properties

Various inflammatory cytokines are upregulated in hyperglycemic milieu [81,82]. Independent of glucose lowering effects, SGLT2i via caspase-1 pathway inhibits secretion of IL-1beta by macrophages, thereby reducing inflammation and fibrosis [83,84,85]. Empagliflozin studies by Panchapakesan et al. on proximal tubular cells demonstrated decreased expression of Toll-like receptor 2 and 4, and NF-kB, further reducing inflammation and subsequent fibrosis [86]. Recently, SGLT2i has demonstrated reduction in interstitial fibrosis along with protection against ischemia-reperfusion injury by upregulating vascular endothelial growth factor (VEGF)-A in a mice model [87].

### 3.5. Cortical Hypoxia Reduction

SGLT2i modulate alterations in oxygen consumption and, therefore, are able to reduce renal cortical hypoxia. It has been noticed that decreased activity of Na-K-ATPase and reduced accumulation of intracellular glucose and sodium contribute to decreased oxygen utility. Fibroblasts might recover erythropoietin production as cortical hypoxia improves [76]. Recent studies have also reported inhibition of HIF-1α expression and related target genes (VEGF, GLUT1, etc.) by SGLT2i along with subsequent reduction in interstitial fibrosis in diabetic kidneys [88].

## 4. Proposed Cardioprotective Mechanisms of SGLT2i

As discussed above, SGLT2i can cause natriuresis, and account for 7% of plasma volume reduction, leading to a decrease in preload [89]. In a meta-analysis, which included 34 randomized controlled studies, SGLT2i increased HDL cholesterol and reduced serum triglycerides [90]. SGLT2i reportedly exhibit significant cardiac benefits similar to renoprotection by lowering body weight, optimizing lipid panel and blood pressure control, improving endothelial function, and reducing arterial stiffness [19]. Apart from described hemodynamic effects, SGLT2i contributes to direct myocardial benefits by promoting lipolysis and ketogenesis [91]. It has been hypothesized that myocardial ketone oxidation could explain the cardioprotective effect of empagliflozin in diabetic patients [92,93]. The proposed mechanism emphasizes utilization of ketone bodies as the preferred heart muscle fuel. Subsequently, this leads to maintenance of mitochondrial integrity and decrease in generation of reactive oxygen species. However, more in vivo studies are needed to determine the exact role of ketone oxidation on cardioprotective effect of empagliflozin.

**Figure 1:**
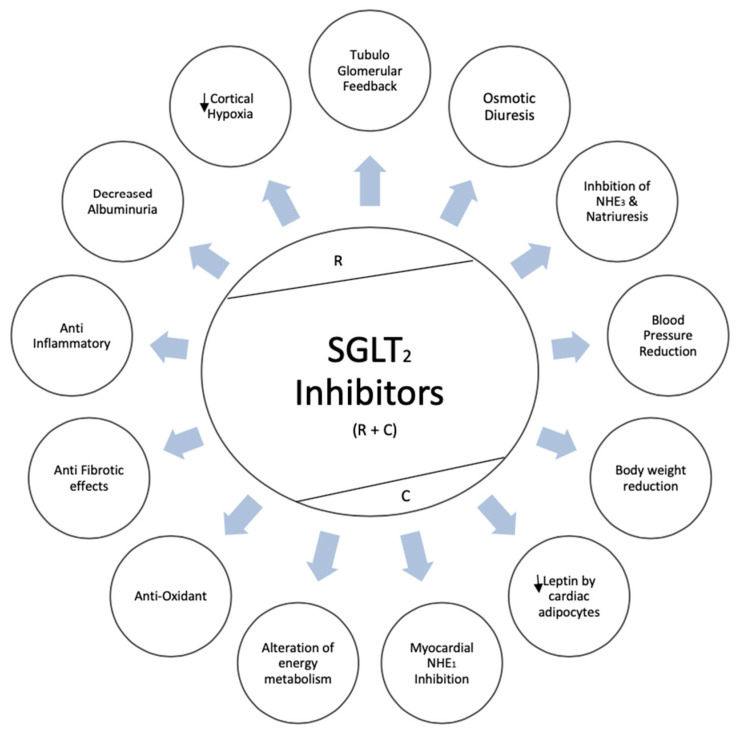
Renoprotective and cardioprotective mechanisms of sodium-glucose co-transporter 2 (SGLT2) inhibitors. R = renoprotective; C–predominantly cardioprotective; R + C- renoprotective and cardioprotective; 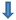 - decreased.

SGLT2i also inhibits NHE1 in the myocardium and lower intracellular sodium and calcium content leading to reduced pro-oxidant and pro-thrombotic state as reported in empagliflozin experimental models [94]. SGLT2i potentiate natriuresis in heart failure patients by inhibiting NHE3 in proximal tubule [95]. In studies by Garvey et al., canagliflozin led to 25% reduction of serum leptin levels, which manifest pro inflammatory properties and a 17% increase of serum adiponectin, which demonstrates anti-inflammatory properties, as compared to glimepiride [96]. Epicardial fat plays a crucial role in heart failure patients and, reportedly, was reduced by dapagliflozin in experimental models in a study by Sato et al. [97]. Finally, the anti-fibrotic effects of SGLT2i manifest through inhibition of myofibroblast differentiation and reduction in pro-inflammatory cytokines, thereby reducing left ventricle mass and improving ejection fraction [98]. Renoprotective and cardioprotective mechanisms of SGLT2i are illustrated in Figure 1.

## 5. Conclusions

SGLT2i has revolutionized treatment approach in patients with type 2 diabetes mellitus. Although primarily glucosuric agents, their renoprotective property appears to be independent of its glucose-lowering effects. Even though multiple randomized controlled studies have illustrated beneficial effects of SGLT2i regarding renal outcomes, a certain degree of hesitation still exists in prescribing combination therapy with SGLT2i in insulin naive diabetic patients. Randomized controlled studies to evaluate therapeutic benefits and renal outcomes of combination therapies as initial approach in patients with type 2 diabetes might further guide the process of establishing more organized treatment approach. Additionally, further controlled studies are required in evaluating the potential role of SGLT2i in reducing proteinuria in non-diabetic CKD patients.

## Figures and Tables

**Table 1:** Randomized placebo-controlled trials demonstrating the treatment outcomes of SGLT2 inhibitors vs. placebo in type 2 diabetes mellitus with CKD.

Clinical Trial	Year	Trial Registration	Total Sample Size	CKD Patients	Kidney Function Inclusion Criteria	Follow-Up	Reported Renal Outcomes
**Canagliflozin**							
CANVAS and CANVAS-R [44,45]	2017 2018	NCT01032629 NCT01989754	10142	2039	eGFR ≥ 30	188 wks	↓ sustained loss of kidney function, eGFR decline, albuminuria, the need for RRT, and death from renal causes
DIA3004 [46]	2014	NCT01064414	269	269	eGFR ≥ 30 and < 50	52 wks	↓ eGFR decline and albuminuria
CREDENCE [43]	2019	NCT02065791	4401	2181	eGFR ≥ 30	2.6 yrs	↓ Renal composite outcomes (ESKD, doubling in SCr, renal or CV death) in both primary and secondary prevention group The trial stopped early due to overwhelming efficacy
**Dapagliflozin**							
MB102029 [47]	2014	NCT00663260	252	252	eGFR ≥ 30 and < 60	104 wks	↓ eGFR decline, albuminuria, and hyperkalemia Slight drop in eGFR during drug initiation
DERIVE [48]	2018	NCT02413398	321	321	eGFR ≥ 45 and < 60	24 wks	↓ Renal related adverse events Slight drop in eGFR during drug initiation
DECLARE-TIMI 58 [22]	2018	NCT01730534	17160	1265	CrCl ≥ 60 mL/min	4.2 yrs	↓ Renal composite outcomes ↓ Acute kidney injury
DAPA-HF [49]	2020	NCT03036124	4724	N/A	eGFR ≥ 30	3 yrs	↓ Doubling in SCr
DIAMOND [50]	2020	NCT03190694	53	33	eGFR ≥ 25	6 wks	No effect on proteinuria reduction in CKD without diabetes Reversible decline in eGFR noted
**Empagliflozin**							
EMPA-REG OUTCOME [21]	2015	NCT01131676	7020	1819	eGFR ≥ 30	3.1 yrs	↓ eGFR decline, and renal composite outcomes
EMPA-REG METSU [51]	2013	NCT01159600	666	58	eGFR ≥ 30	24 wks	↓ Renal composite outcomes
EMPA-REG RENAL [52]	2014	NCT01164501	738	448	eGFR ≥ 15	52 wks	↓ Renal composite outcomes
Halden, et al. [53]	2019	NCT03157414	44	44 (KTx)	eGFR ≥ 30	24 wks	↓ eGFR within 8 weeks of treatment No change in eGFR from 8-24 weeks
**Bexagliflozin**							
Allegretti, et al. [54]	2019	NCT02836873	312	312	eGFR ≥ 30 and < 60	24 wks	↓ albuminuria Study not designed to evaluate the impact on long-term kidney disease

Abbreviations: SGLT2-sodium-glucose co-transporter 2; CKD—chronic kidney disease; CrCl—creatinine clearance; CV—cardiovascular; eGFR—estimated glomerular filtration rate; ESKD—end-stage kidney disease; KTx—kidney transplant; RRT—renal replacement therapy; SCr—serum creatinine.

**Table 2:** Trials demonstrating the treatment outcomes of SGLT2 inhibitor in non-diabetics with chronic kidney disease.

Clinical Trial	Year	Trial Type	Total Sample Size	Kidney Function Inclusion Criteria	Follow-Up	Reported Renal Outcomes
**Dapagliflozin**						
Zhang et al. [55]	2016	Pre-clinical	53	Subtotal Nephrectomized rats.	12 weeks	No improvement in proteinuria, tubulointerstitial fibrosis or eGFR.
Cassis et al. [56]	2018	Pre-clinical	37	Non-diabetic proteinuric mice, unilateral nephrectomy.	23 days	Decrease in podocyte damage, reduction in proteinuria
Jaikumkao et al. [60]	2018	Pre-clinical	24	Obese prediabetic rats	4 weeks	Decrease in podocyte damage, reduction in proteinuria
Rajasekeran et al. [58]	2018	Clinical	10	Biopsy proven FSGS, eGFR > 45mL/min, proteinuria 30 mg-6 gr	5 weeks	No effect on bodyweight, eGFR or proteinuria
DIAMOND [50]	2020	Clinical	53	eGFR ≥ 25	6 weeks	No effect on proteinuria reduction in CKD without diabetes Reversible decline in eGFR noted
**Empagliflozin**						
Ma et al. [57]	2017	Pre-clinical	20	CKD mice	7–14 days	No reno-protective benefit
**Canagliflozin**						
Bays et al. [59]	2014	Clinical	376	Non-diabetic obese patients, BMI 30-50	12 weeks	No renal benefit

Abbreviations: SGLT2-sodium-glucose co-transporter 2; CKD—chronic kidney disease; eGFR—estimated glomerular filtration rate; FSGS—focal segmental glomerulosclerosis; BMI—body mass index.
